# Anti-PEG Antibodies and Their Biological Impact on PEGylated Drugs: Challenges and Strategies for Optimization

**DOI:** 10.3390/pharmaceutics17081074

**Published:** 2025-08-20

**Authors:** Shujun Fu, Xueran Zhu, Fanghua Huang, Xiaoyan Chen

**Affiliations:** 1 Center for Drug Evaluation, Chinese National Medical Product Administration, Beijing 100076, China; fushj@cde.org.cn; 2 State Key Laboratory of Drug Research, Shanghai Institute of Materia Medica, Chinese Academy of Sciences, Shanghai 201203, China; zhuxueran@simm.ac.cn

**Keywords:** anti-PEG antibodies, pre-existing anti-PEG antibodies, immunogenicity, ABC effect, biological effects

## Abstract

Polyethylene glycol (PEG) has been widely utilized in optimizing therapeutics due to its excellent biocompatibility and chemical stability. However, multiple dosing of PEGylated drugs may result in toxicity due to PEG accumulation in tissues, leading to the formation of anti-PEG antibodies (APAs), which can accelerate drug clearance, reduce efficacy, and alongside enhanced side effects, such as allergic reactions. Notably, pre-existing APAs have also been detected in individuals with no prior exposure to PEGylated drugs, raising additional clinical concerns. This review summarizes the mechanisms of APA generation, the factors influencing PEG immunogenicity, and the biological consequences of APAs on drug pharmacokinetics, efficacy, and safety. We also discuss current challenges in APA detection and highlight strategies to minimize immunogenic responses, including PEG modification, immunomodulation, and alternative polymers. This review aims to provide a comprehensive reference for the rational design, evaluation, and clinical management of PEGylated drugs.

## 1. Introduction

Polyethylene glycol (PEG) is a polymer that can covalently bond to the active sites of drug molecules or the surfaces of drug delivery carriers, creating PEGylated drugs or delivery systems [1]. The U.S. Food and Drug Administration (FDA) has approved 41 PEGylated drugs, including two PEGylated small-molecule drugs, five PEGylated nanomedicines, and 34 macromolecular PEGylated medicines, such as enzymes, antibodies, and antibody fragments [2,3,4,5,6].

PEGylation modifies the physicochemical properties of drugs, including their conformation, electrostatic interactions, and hydrophobicity. This alteration enhances the drug’s pharmacokinetic, pharmacodynamic, and immunological characteristics, broadening their clinical applicability [7]. A protective shield can be created by attaching PEG chains to drug molecules. This modification effectively reduces renal filtration and uptake by the mononuclear phagocyte system (MPS), thus prolonging the circulation of the drug in the body. As a result, the drug is gradually released at the target tissues, ultimately enhancing therapeutic efficacy [8,9]. Moreover, PEGylation enhances the water solubility of protein- and peptide-based therapeutics, reduces their enzymatic degradation and phagocytosis by the reticuloendothelial system (RES), decreases glomerular filtration, and lowers immunogenicity [10].

For some biologics, PEGylation is not merely a strategy to improve pharmacokinetics but a prerequisite for therapeutic viability. Drugs such as L-asparaginase, interferon-α, and uricase exhibit rapid degradation, high immunogenicity, or poor solubility in their native forms, which severely limits their clinical use. PEGylation enables these molecules to maintain systemic circulation, evade immune recognition, and exhibit improved patient outcomes. In such cases, PEGylation is considered essential rather than optional, underscoring its critical role in drug development [11].

PEGylated drugs have demonstrated substantial clinical value in a range of therapeutic areas. For example, peginterferon alfa-2a has shown a prolonged half-life and enhanced antiviral activity in the treatment of chronic hepatitis C, enabling less frequent dosing and improved patient compliance [11]. In oncology, PEGylated liposomal doxorubicin (Doxil^®^) has improved pharmacokinetics and reduced cardiotoxicity compared to conventional formulations, making it a widely used treatment for ovarian cancer and multiple myeloma [12,13]. These clinical successes underscore the therapeutic advantages of PEGylation. However, accumulating evidence of anti-PEG immune responses has brought attention to potential risks, such as reduced efficacy and hypersensitivity reactions, necessitating a deeper understanding of PEG immunogenicity.

Although PEG was classified as “Generally Recognized as Safe” (GRAS) for use as a food additive by the U.S. FDA as early as 1973 [14], the extensive clinical use of PEGylated drugs has resulted in a growing number of reports suggesting that PEG and its derivatives may have immunogenic potential in both animals and humans [6,15,16]. Multiple dosing of PEGylated drugs can lead to the production of anti-PEG antibodies (APAs), which have been linked to decreased therapeutic efficacy and an increased risk of adverse reactions. For instance, in a pediatric trial involving patients with hematologic malignancies who had not previously undergone PEGylated asparaginase treatment [17], three out of four patients experienced hypersensitivity reactions. Additionally, their serum asparaginase activity (SAA) fell below the detection limit by Day 8 post-infusion. Testing via enzyme-linked immunosorbent assay (ELISA) confirmed the presence of anti-PEG IgG antibodies in the serum of three reported hypersensitive patients. The blood APA significantly accelerated the clearance of PEGylated drugs, known as accelerated blood clearance [18,19].

In addition to pharmaceutical applications, PEG and its derivatives are widely utilized in cosmetics, personal care products, and food additives. This may account for the presence of pre-existing APAs found in individuals who have never received PEGylated therapies [20]. In 1984, Richter and colleagues [21] reported a pre-existing APA detection rate of approximately 0.2% in 453 healthy volunteers from Japan, Germany, and Italy using a passive hemagglutination method. They considered this finding to have no clinical significance for PEGylated therapies.

The landscape regarding APAs has changed significantly in the 21st century. In 2016, Yang et al. [22] reported a 72% rate of detecting pre-existing APAs in a study involving 377 healthy individuals using a standardized ELISA with chimeric anti-PEG monoclonal antibodies. This rate was significantly higher than the 0.2–44% range found in earlier studies. The researchers attributed this discrepancy mainly to the limited sensitivity of previous testing methods and suggested that pre-existing APAs may be more widespread in humans than previously thought.

Both drug-induced and pre-existing APAs can result in a variety of biological effects in vivo. The extent to which these antibodies affect therapeutic effectiveness and contribute to adverse reactions is still not fully understood. Additionally, there are significant challenges in detecting APAs, which adds further uncertainty and risk to the development and clinical use of PEGylated drugs.

This review summarizes the mechanisms behind APA generation and the factors that affect the immunogenicity of PEG. It focuses on the biological effects of APAs on PEGylated drugs in vivo, the methods for APA detection, and strategies to mitigate the impact of APAs. The goal is to serve as a useful reference for the research, evaluation, and rational design of PEGylated therapeutics.

## 2. Mechanisms Underlying Anti-PEG Antibody Generation and Key Immunogenicity Factors

### 2.1. Mechanisms of Anti-PEG Antibody Generation

#### 2.1.1. Thymus-Dependent Antigen Response

PEGylated therapeutics, which include proteins, peptides, small molecules, and liposomal drugs, can trigger APA responses via the classical T cell-dependent pathway. When B cell receptors (BCRs) bind specifically to the PEG backbone, B cells are activated and differentiate into plasma cells, secreting anti-PEG IgM antibodies. However, the production of anti-PEG IgG antibodies requires signals from CD4^+^ T cells, specifically follicular helper T (TFH) cells located in secondary lymphoid organs. The interaction of CD40 ligand on TFH cells with B cells triggers the expression of activation-induced cytidine deaminase (AID) [23]. B cells take up large quantities of PEGylated drugs and express the major histocompatibility complex (MHC)-bound antigens on their surface, aiding interaction with TFH cells. While B cells recognize the PEG moiety and secrete APAs, TFH cells respond to the non-human therapeutic protein components of PEGylated drugs, triggering an immune response. This explains the mechanism underlying thymus-dependent APA production.

#### 2.1.2. Thymus-Independent Antigen Response

In animal models, B and T cells can initiate immune reactions against PEGylated human proteins by identifying them as foreign molecules. Non-protein antigens, known as thymus-independent antigens, can induce antibody responses, such as the secretion of IgM and IgG antibodies. These antigens include nucleic acids, empty liposomes (including liposomes encapsulating cytotoxic drugs), nanoparticles, and nucleic-acid-based nanocarriers. In contrast to peptide-MHC class II antigens, thymus-independent type 2 (TI-2) antigens demonstrate multivalency, enabling them to cross-link BCRs on the cell surface. Linear PEG polymer chains have multiple epitopes that can bind antibodies, which qualifies PEG as a TI-2 antigen. Marginal zone B cells play a primary role in antibody responses to TI-2 antigens. The cross-linking of BCRs initiates the activation signal. At the same time, innate immune cells can provide extra stimulatory signals through toll-like receptors (TLRs), which include essential B cell activators from the tumor necrosis factor (TNF) superfamily [24]. As a result of exposure to thymus-independent antigens, murine marginal zone B cells primarily produce IgM, IgG2b, IgG3, and IgA, while human marginal zone B cells mainly secrete IgM, IgG1, IgG2, and IgA2.

**Figure 1:**
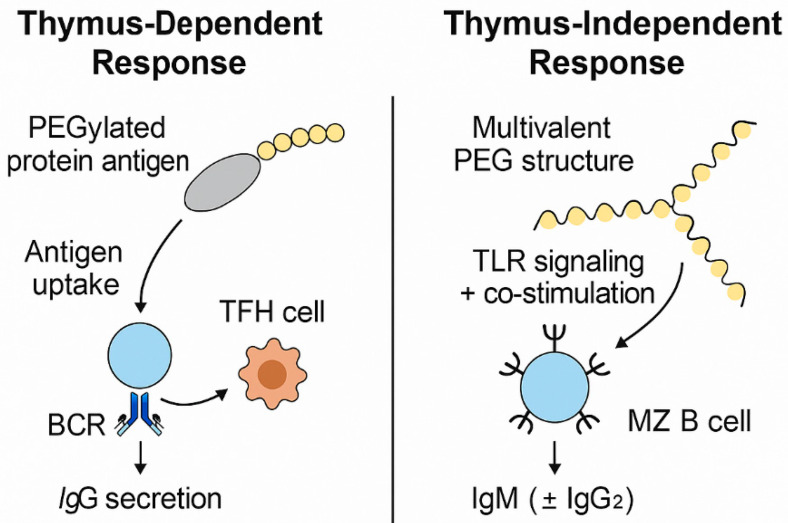
Mechanisms of anti-PEG antibody generation. The thymus-dependent response involves antigen processing by B cells and interaction with follicular helper T (TFH) cells, leading to IgG production. In contrast, thymus-independent responses are initiated by multivalent PEG structures that directly activate marginal zone (MZ) B cells, typically resulting in IgM (±IgG_2_) secretion. Created by the authors.

In summary, thymus-dependent immune responses involve the cooperation of B cells and helper T cells (particularly TFH cells), typically leading to high-affinity, class-switched antibodies such as IgG. These responses are triggered by complex protein-containing antigens, including PEG-protein conjugates. In contrast, thymus-independent responses are predominantly mediated by marginal zone B cells or B1 cells and do not require T cell help. Thymus-independent responses are typically elicited by highly repetitive structures, such as the linear PEG backbone, and mainly generate IgM (and low-affinity IgG2 in humans). These mechanisms differ in their cellular pathways, antigen recognition profiles, and antibody outputs, as illustrated in Figure 1.

### 2.2. Polymer- and Carrier-Related Factors Affecting PEG Immunogenicity

**Figure 2:**
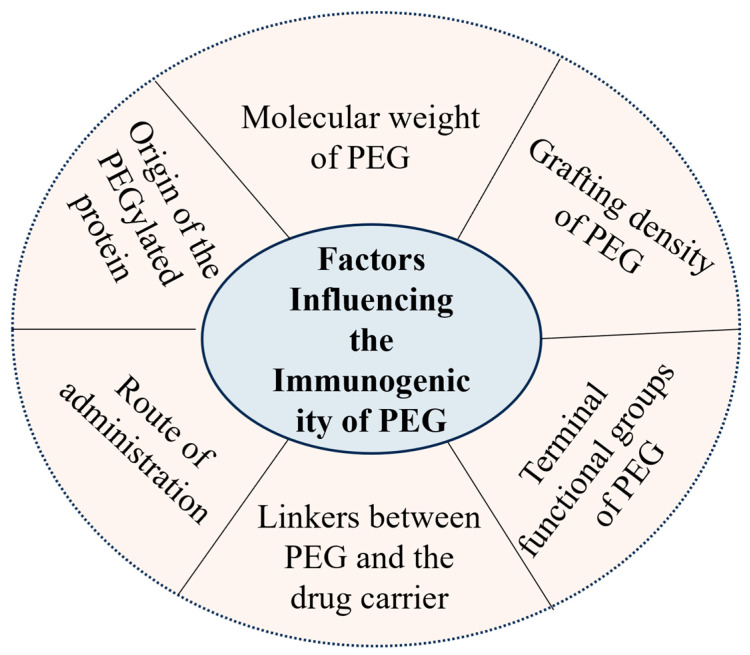
Factors influencing the immunogenicity of PEG. PEG immunogenicity is affected by various physicochemical and structural features, including PEG molecular weight, terminal functional groups, surface density, and the nature of the conjugated carrier. Route of administration and dosing frequency also impact antibody generation risk.

Free PEG molecules typically exhibit minimal immunogenicity. However, PEG conjugated to proteins, peptides, small molecules, or liposomal carriers can become immunogenic. The antibody response is directed to the PEG moiety and can target various structural components of the entire PEGylated construct. As a result, the immunogenicity of PEGylated therapeutics depends on multiple characteristics of the polymer and its carrier system, as illustrated in Figure 2, which summarizes the major factors affecting PEG immunogenicity.

Firstly, the physicochemical properties of PEG molecules, such as molecular weight, grafting density, and terminal functional groups, can significantly influence their immunogenicity [25]. PEGs with higher molecular weights exhibit increased immunogenicity. Bovine serum albumin (BSA) modified with PEG 30,000 and ovalbumin (OVA) modified with PEG 20,000 induced significantly stronger in vivo anti-PEG IgM responses than their counterparts modified with PEG 2000 and 5000, respectively. Although PEG 2000 and PEG 5000 are commonly used in PEGylated therapeutics due to their favorable balance between solubility, hydrodynamic shielding, and circulation half-life, lower molecular weight PEGs such as PEG 500 and PEG 1000 have also been investigated, particularly in small-molecule conjugates and prodrugs [26]. These shorter chains provide limited steric hindrance and are rapidly cleared via the kidneys, making them less suitable for sustained delivery but potentially useful in local or short-acting applications.

A study quantitatively analyzed the levels of anti-PEG IgG, IgM, and IgE antibodies in blood samples from a clinical trial involving administration of Comirnaty^®^, a COVID-19 vaccine based on PEGylated lipid nanoparticles [27]. Among 78 participants who received mRNA lipid nanoparticles (LNPs), levels of IgG were significantly elevated three weeks after injection. In contrast, IgM levels were lower, and IgE remained undetectable. When comparing LNPs modified with a low PEG density of 0.3 mol% DSPE-PEG 2000 to Comirnaty^®^, which contained 1.5 mol% PEG, a notably reduced affinity for APAs was exhibited. The secretion of APAs showed a relationship with the concentrations of surface PEG in the LNP formulation [28].

Because various PEG structures contain similar, repeating ethylene glycol units, APAs triggered by one type of PEG may show cross-reactivity with others. Sherman et al. [29] reported that APAs generated by hydroxy-PEG-conjugated proteins displayed similar affinity for methoxy-PEG and hydroxy-PEG. In contrast, antibodies induced by methoxy-PEG-conjugated proteins have a higher specificity for methoxy-PEG than for hydroxy-PEG. The binding affinities of antibodies formed with various PEG terminal groups rank as follows: hydroxyl (-OH) < amino (-NH_2_) < methoxy (-OCH_3_) < butoxy (-O-(CH_2_)_3_CH_3_) < tert-butoxy (-O-(CH_3_)_3_).

Secondly, the dosage and frequency of PEGylated drug administration are key influencing factors. Elsadek et al. [30] found that subcutaneous injection of Pegasys (PEGylated interferon alfa-2a) in mice at a dose of 3 μg/kg did not elicit negligible anti-PEG immune responses. After administering three to four repeated 150 μg/kg doses, anti-PEG IgM became detectable. Furthermore, the route of administration is closely linked to the immunogenicity of PEG. Intravenous injections are more likely to trigger a systemic immune response, while subcutaneous injections tend to elicit localized immune reactions primarily. Elsadek et al. [31] showed that administering PEG-G-CSF (PEGylated granulocyte colony-stimulating factor) intravenously or subcutaneously at doses greater than 0.06 mg/kg in mice produced anti-PEG IgM antibodies. This response was dose-dependent, with higher antibody levels observed following intravenous delivery than subcutaneous injection.

Finally, the linker between PEG and the drug carrier influences immunogenicity [9]. Poppenborg et al. [32] demonstrated that both amide and succinyl linkages between PEG and asparaginase could induce similar levels of anti-PEG antibody production after administration of PEG-asparaginase.

The origin of the PEGylated protein significantly affects the induction of APA responses. During antibody maturation and affinity differentiation within germinal centers, PEGylated proteins derived from human sources typically induce fewer anti-PEG immune responses in patients. The relatively low incidence of APAs is reported against human-derived PEGylated factor VIII (FVIII) [33]. In contrast, PEGylated non-human sources trigger much stronger anti-PEG immune responses. Most commercially available PEGylated proteins are based on non-human sources, raising significant concerns regarding PEG-related immunogenicity.

## 3. Biological Consequences of Anti-PEG Antibodies on Drug Efficacy and Safety

The binding of APAs to PEGylated drugs initiates a cascade of biological effects, presenting dual challenges to clinical treatment. On one hand, APAs alter the pharmacokinetic behavior of the drug, leading to diminished therapeutic efficacy. This involves a significant acceleration of systemic clearance and a decrease in the area under the plasma concentration–time curve, which reduces the drug’s bioavailability. The accelerated blood clearance (ABC effect) may lead to the rapid elimination of the drug by the immune system before it can exert its intended therapeutic effect. On the other hand, APAs may induce hypersensitivity reactions (HSRs), a type of pseudoallergic response, ranging from mild symptoms such as skin rashes to severe, life-threatening anaphylactic shock.

### 3.1. Impact on Pharmacokinetics: Accelerated Blood Clearance (ABC) Phenomenon

Repeated intravenous administrations of complex PEGylated proteins or nanoparticles in both animals and humans trigger the ABC phenomenon, leading to a reduced circulation time of PEGylated drugs and, as a result, a decrease in therapeutic efficacy. The ABC phenomenon was first described by Dams et al. [34], who observed that the plasma elimination half-life of radiolabeled PEGylated liposomes in Wistar rats was 2.4 h after the initial injection. However, when a second injection was administered 5 to 21 days later, the half-life significantly decreased to just 0.1 h.

Further supporting evidence shows the clinical and preclinical significance of this phenomenon. Hsieh et al. [35] reported that mice with pre-existing APAs exhibited significantly reduced tumor accumulation of PEGylated liposomal doxorubicin (LipoDox), resulting in diminished antitumor efficacy compared to APA-negative controls. Armstrong et al. [17] observed that leukemia patients with anti-PEG antibodies experienced rapid loss of serum asparaginase activity when treated with PEG-asparaginase, suggesting antibody-mediated clearance. These findings provide quantitative and mechanistic validation of the ABC effect and highlight the importance of monitoring APA levels in both preclinical and clinical settings.

**Figure 3:**
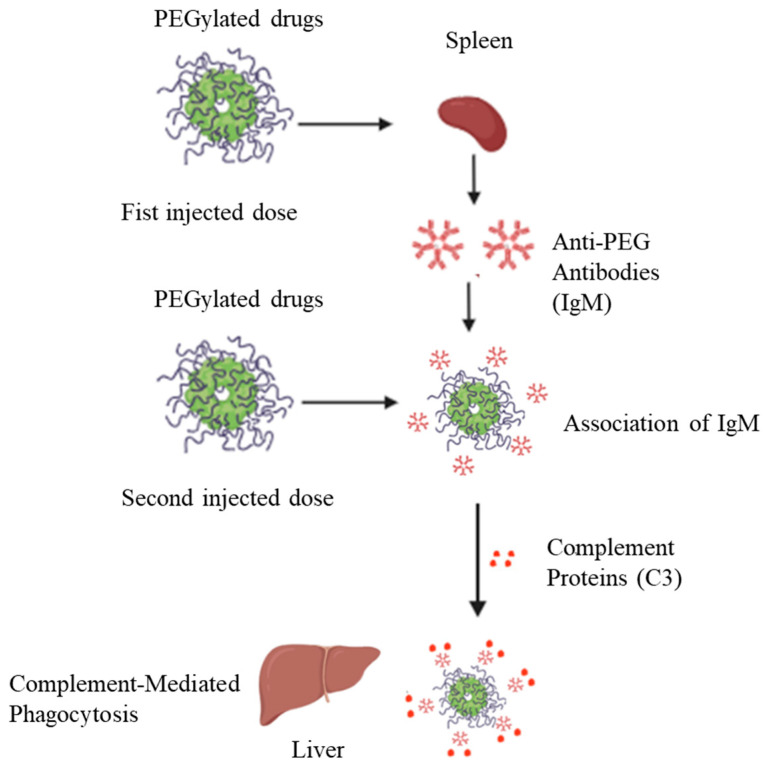
Formation mechanisms of anti-PEG antibodies and the underlying mechanisms of the ABC phenomenon (modified from Ref. [40]). The diagram illustrates how PEGylated drugs can induce either thymus-dependent or thymus-independent immune responses, resulting in anti-PEG antibody (APA) production. Upon repeated dosing, APA—particularly IgM—binds to PEGylated drugs, activates the classical complement pathway, and leads to opsonization and rapid clearance by Kupffer cells. This immune cascade contributes to reduced circulation time and diminished drug efficacy.

Laverman et al. [36] reported that the ABC phenomenon has an induction phase, during which the biological system is “primed” after the first administration, and an effectuation phase, where the PEGylated drug is rapidly cleared upon subsequent administration. In initial administration, PEGylated drugs interact with surface immunoglobulins on reactive B cells in the spleen, leading to the T cell-independent production of anti-PEG IgM. These IgM antibodies persist in the circulation at the second dosing and bind to the drug’s PEG moiety, activating the classical complement pathway. This leads to opsonization via complement fragment C3, followed by phagocytosis mediated by complement receptors on Kupffer cells, which speeds up the drug’s clearance from the bloodstream (Figure 3).

The levels of anti-PEG IgM secretion are linked to the intensity of the ABC phenomenon. In rat models with depleted Kupffer cells, repeated dosing of PEGylated drugs can further stimulate marginal zone B cells in the spleen, leading to increased IgM production and a stronger ABC response [37]. Zhao et al. [38] demonstrated that the ABC phenomenon is also observed after subcutaneous injection of PEGylated liposomes, proposing that regional lymph nodes and associated lymphocytes have a key role in initiating immune responses and mediating the generation of antibodies against PEGylated liposomes. The ABC phenomenon from PEGylated drugs is complex. Further investigation is needed to clarify contributing factors and the specific tissues and cell types involved.

APA generation can affect the distribution of drugs in vivo. Binding the antibodies to PEGylated medications will alter the distribution profile of these drugs, leading to faster clearance from blood and accumulation in the liver and spleen [39]. The enhanced distribution can raise drug concentrations locally, thereby increasing the risk of organ-specific toxicity. Dams et al. [34] also demonstrated the increased accumulation of radiolabeled PEGylated cholesteryl liposomes in the liver and spleen after a second injection, four hours after the first. Hepatic accumulation increased from 8.1 ± 0.8% of the injected dose at the first dose to 46.2 ± 9.8% at the second (*p* < 0.01). However, splenic accumulation increased from 2.2 ± 0.2% to 5.3 ± 0.7% (*p* < 0.01). Such a change in the pharmacokinetics of PEGylated liposomes suggested a potential clinical safety concern. If the encapsulated drug is cytotoxic, aggregation of liposomes with hepatic Kupffer cells may lead to apoptosis and necrosis of these cells. Recovery of Kupffer cell function takes about two weeks, during which the presence of bacteremia could be fatal for cancer patients [37].

### 3.2. Pharmacodynamic Consequences and Therapeutic Limitations

The accelerated clearance of PEGylated drugs by APAs leads to a decrease in effective drug concentration in circulation, which directly undermines therapeutic efficacy. This is especially crucial in cancer treatment, as inadequate drug levels can result in poor tumor control [40]. In a study involving PEGylated interferon for treating hepatitis C [41], it was found that patients with elevated APA levels showed significantly reduced antiviral interferon efficacy. Hsieh et al. [35] reported that pre-existing APAs altered the pharmacokinetics of liposomal doxorubicin (LipoDox), reducing drug accumulation in the tumors, leading to diminished outcomes. In contrast to APA-negative mouse models, mice displaying pre-existing APAs exhibited a significantly reduced response to LipoDox, emphasizing the importance of accounting for antibody levels when administering PEGylated drugs.

The presence of APAs may also narrow the therapeutic window of PEGylated drugs and affect the drug’s efficacy by reducing patients’ exposure. Accordingly, to ensure therapeutic efficacy, multiple doses may be required, which can increase both healthcare costs and the risk of side effects, thereby negatively impacting patients’ quality of life. Consequently, optimizing dosing regimens to prevent or mitigate APA development has become critical to clinical management strategies [42].

### 3.3. Immunological and Hypersensitivity Risks

The most frequently observed adverse effects of PEGylated drugs include reduced bioactivity and hypersensitivity reactions, as well as chronic inflammation caused by prolonged immune system overactivation [42]. Although anti-PEG IgG and IgM are the most frequently studied subtypes, recent studies have suggested the possible involvement of anti-PEG IgE in hypersensitivity reactions. Although rare, IgE-mediated responses can trigger severe anaphylactic reactions upon exposure to PEGylated compounds. For instance, some reports have identified low levels of anti-PEG IgE in patients experiencing immediate allergic responses to PEG-containing COVID-19 mRNA vaccines [43]. Further studies are needed to elucidate the prevalence, clinical implications, and detection methods of anti-PEG IgE.

When bound to PEGylated drugs, APAs activate the complement system, initiating a series of immune responses. This activation encourages the release of allergic mediators, including histamine, leukotrienes, platelet-activating factors, and tryptase. These inflammatory mediators subsequently interact with effector cells, particularly in the cardiopulmonary system, leading to symptoms of hypersensitivity reactions [44]. In 1984, Rachel et al. [45] reported that 75% of children with acute lymphoblastic leukemia developed hypersensitivity reactions and produced APAs following intravenous administration of PEGylated Erwinia asparaginase, leading to a rapid loss of enzymatic activity. Similarly, reports indicate that up to 45% of cancer patients treated with Doxil^®^ experienced hypersensitivity reactions when not premedicated. However, this incidence decreased significantly to 4.0–7.1% when patients received antihistamines before treatment [46].

Beyond allergic responses, the safety of PEGylated nanomedicines is also of concern due to premature drug leakage. These nanomedicines are structurally complex and composed of diverse materials, and the delivery systems may provoke unexpected and intricate immune responses. Shiraishi et al. [42] evaluated the toxicity of polymeric micelles made from PEG-b-polyaspartate block copolymers in Donryu rats. The number of foam cells in the lungs and lymph nodes increased after five intravenous injections of 20 mg/kg and significantly increased in the spleen with 200 mg/kg. The formation of foam cells often indicates toxicity or tissue damage caused by the drug or carrier. Moreover, there was a significant rise in CD68-positive macrophages in the treated rats’ spleen, liver, and lungs.

### 3.4. Species Differences in Anti-PEG Antibodies and Their Implications for Clinical Translation

The production of APA is associated with the immunological characteristics of different species. For decades, nonclinical studies have focused on exploring the potential impact of APAs on PEGylated nanomedicines. The immunogenicity of PEGylated drugs and excipients has been extensively researched in rodents. For instance, poloxamer 188 (F68), which contains poly (ethylene oxide) segments structurally similar to PEG, significantly induced anti-PEG IgM and IgG in mice. This immune response can lead to the ABC effect upon subsequent administration of PEGylated nanodrugs [47].

Importantly, clinical trials indicated that APAs were found in healthy individuals not exposed to PEGylated drugs, and the prevalence rates increased over time. This trend may be attributed to frequent exposure to PEG-containing pharmaceutical excipients in everyday products, such as toothpaste and cosmetics [20]. Preclinical models may not fully represent the complexity of human exposure; however, they are still valuable for understanding the mechanisms of adverse events. These insights highlight the significance of avoiding highly immunogenic excipients in clinical settings.

The preclinical animal models play a critical role in evaluating the impact of APAs on drug metabolism and efficacy and assessing APAs on the pharmacokinetics and therapeutic outcomes of PEGylated drugs. For instance, studies on rat PK have demonstrated that the presence of APAs significantly reduced the drug’s half-life. Additionally, competitive ELISA was employed to quantify antibody levels and provide a valuable reference for dosage adjustment in clinical settings. The porcine species, which possesses a complement system highly homologous to that of humans, has been utilized to investigate PEG-specific allergic reactions. Research has shown that free PEG can reduce the risk of pseudoallergic shock caused by PEGylated liposomes.

Furthermore, repeated use of free PEG does not increase antibody levels. This information provides a scientific foundation for premedication strategies to minimize hypersensitivity in clinical settings [48]. However, interspecies differences in immune responses must be interpreted with caution. For instance, IgM in mouse models primarily mediates the ABC effect. In contrast, IgG predominates in humans, suggesting preclinical findings should be adjusted according to species-specific immunological characteristics [49].

Studies should guide the selection of animal models to predict clinical outcomes. Rodent models are cost-effective and allow for investigating short-term effects. They are suitable for initial screening of PEGylated drug immunogenicity, pharmacokinetics, and organ accumulation. Jiang K et al. used a mouse model to demonstrate that hepatic uptake of PEGylated liposomes, mediated primarily by Kupffer cells. The finding was consistent with humans and indicated hepatic accumulations exacerbated by anti-PEG antibodies [49].

Large animals, such as pigs, can be used to investigate severe allergic reactions or organ-specific toxicities due to their closer physiological and immunological similarity to humans. Shen LM et al. [48] successfully used a complement activation-related pseudoallergy (CARPA) pig model for estimating PEG-mediated allergic reactions with a sensitivity 1000 times greater than that of rodents. The CARPA pig model has also been validated to assess the suppressive effects of free PEG on hypersensitivity induced by PEGylated liposomes. Non-human primates, such as monkeys, have immune systems that closely mimic those of humans, making them suitable for evaluating the long-term effects of APAs on therapeutic efficacy and the cross-species immunogenicity of PEGylated drugs.

## 4. Analytical Techniques for Anti-PEG Antibody Detection: Sensitivity and Limitations

The passive hemagglutination assay (PHA) was the first documented method for detecting APAs. While quick and straightforward, its low sensitivity and lack of precise quantification have decreased usage. Various techniques have been used to enhance sensitivity detection, including Western blot (WB), Acoustic Membrane Microparticle technology (AMMP), Enzyme-Linked Immunosorbent Assay (ELISA), and Flow Cytometry. These methods can amplify signals through enzymatic or fluorescent labeling; still, they are semi-quantitative, with limitations that depend on assay conditions.

Technologies such as Surface Plasmon Resonance (SPR), Meso Scale Discovery (MSD), and Dual Cytometric Bead Assay (DCBA) provide ultra-sensitive, rapid, and accurate quantification capabilities, facilitating both screening and quantitative measurement of APAs (IgG and IgM) in human blood samples. The high cost of specialized instruments and reagents remains a limitation to their extensive application. Table 1 lists commonly used APA detection methods and their advantages and disadvantages in the order of increasing sensitivity.

Most modern techniques, except PHA, can differentiate between APA subtypes. Currently, there is a shortage of human-derived or humanized APA-positive control antibodies. Chimeric antibodies created through DNA technology are commonly used as standards for ELISA and electrochemiluminescence assays [34,36,50]. ELISA is currently regarded as the gold standard for APA detection, because the binding activity of APAs to PEGylated drugs is more clinically relevant than their absolute concentration. This is because binding affinity directly determines the biological impact of APAs on PEGylated therapeutics. In addition, ELISA enables the measurement of various binding patterns by adjusting the coating reagent, such as the affinity of APAs for PEG with different terminal groups.

Currently, mainstream ELISA protocols use standardized chimeric human anti-PEG monoclonal antibodies to quantify APA concentrations. Compared to conventional ELISA, newer technologies, such as AMMP, SPR, MSD, and DCBA, require less sample volume, allowing for simultaneous analysis of various sample components, and offer broader detection ranges and faster processing speeds. With continuous cost reductions and increasing accessibility of instrumentation, these emerging technologies are expected to surpass traditional ELISA in the field of APA detection.

**Table 1:** Detection techniques for anti-PEG antibodies.

Detection Techniques	Detection Principle	Sensitivity Range	Advantages	Disadvantages	Ref.
Passive hemagglutination assay (PHA)	The surface of red blood cells is modified with methoxy-PEG (or other PEG derivatives) and their incubation with serial dilutions of the test serum. In the presence of anti-PEG antibodies, agglutination of red blood cells occurs, which can be observed visually or measured using a spectrophotometer.	1–10 µg/mL	Relatively rapid, cost-effective detection method, and suitable for high-throughput screening	Low sensitivity, in ability to differentiate antibody isotypes	[19,20]
Western blot (WB)	Following incubation of dye-labeled PEGylated antigens (e.g., PEGylated liposomes) with the serum sample, antibody–antigen complexes are enriched through gel filtration. The dye-containing fractions are subjected to SDS-PAGE and transferred to a nitrocellulose membrane. After recognition by conjugated anti-IgG/IgM antibodies, anti-PEG antibodies are identified and visualized via enzyme-linked immunosorbent assay.	0.5–5 µg/mL	Physiological interaction between PEG and anti-PEG antibodies, differentiation of antibody subtypes	Technically complex, consisting of multiple steps, providing only qualitative or semi-quantitative analysis	[51]
Acoustic Membrane Microparticle technology (AMMP)	The sample is diluted and incubated with methoxy-PEG-coated microparticles to capture anti-PEG antibodies. The resulting complexes are immobilized on an acoustic membrane coated with protein A. A signal is generated by the mass change on the membrane surface that is proportional to the mass of the anti-PEG antibodies.	1–10 ng/mL (IgG), less for IgM	Lack of non-specific binding, absolute quantification	Unreliable for IgM quantification	[52]
Enzyme Immunosorbent assay (ELISA)	In these assays, the PEG-containing antigen is immobilized in some form within a 96-well plate, capturing anti-PEG antibodies from the test sample. The bound antibodies are detected either by a conjugated antigen (bridging format) or by a detection antibody (or other recognition molecule) and visualized through an enzymatic reaction.	0.01–10 µg/mL	High sensitivity and specificity, reliable and quantitative detection of anti-PEG antibodies, most used quantitative technique for APAs, distinguishing antibody isotypes, not requiring expensive specialized equipment	Relative values rather than absolute concentrations, complex procedures	[51,53,54]
Flow Cytometry	PEG-modified polymers (e.g., TentaGel-OH polystyrene beads) are immobilized as antigens within the sample. Following washing with a suitable buffer, the beads are stained with fluorescent dyes conjugated to anti-IgG or anti-IgM antibodies to detect bound IgG or IgM. The mean fluorescence intensity of the beads is measured using flow cytometry.	0.1–1 µg/mL	High sensitivity, capability of distinguishing between different types of anti-PEG antibodies	Inaccurate reflection of in vivo interactions due to differences in binding conditions, lacking fluorescent standards	[25]
Surface Plasmon Resonance (SPR)	PEGylated polymers are immobilized on an SPR (Surface Plasmon Resonance) sensor chip to capture APAs from samples passing through the sensor unit. The quantity of bound anti-PEG antibodies is determined by measuring the wavelength shift, which corresponds to the mass of the antibodies that adhere to the sensor chip.	1–10 ng/mL	High sensitivity, absolute quantification of anti-PEG antibody concentrations, ability to isotype differentiation	High equipment cost, requirement for preprocessing to remove potential interfering substances	[55]
Meso Scale Discovery (MSD)	The MSD platform utilizes PEG molecules immobilized on electrode surfaces as antigens for detecting anti-PEG antibodies. Upon antigen–antibody binding, an electrochemiluminescent reaction is triggered. The ruthenium-labeled complex emits light at a wavelength of 620 nm, with an intensity of proportional to the concentration of anti-PEG antibodies in the sample.	0.1–10 ng/mL	Minimal sample volume, high detection sensitivity, simultaneously detection of anti-PEG IgM and IgG in samples, faster results, suitable for large-scale screening	High equipment cost, requiring specialized MSD instruments, complex operation	[56]
Dual Cytometric Bead Assay (DCBA)	A multiplex analytical method for detecting anti-PEG antibodies involves covalently binding PEG molecules to fluorescently labeled microspheres. These microspheres are then incubated with the test sample, allowing any anti-PEG antibodies present to attach to the PEG-coated surfaces. The resulting binding is detected through fluorescence using flow cytometry.	<1 ng/mL (as low as 0.1 ng/mL)	High sensitivity and specificity, capability of detecting both anti-PEG IgM and IgG in one sample	High equipment cost, complex technique	[43]

## 5. Current Strategies to Overcome Anti-PEG Antibody Responses in Therapeutic Design

As the number of PEGylated drugs in clinical development and on the market continues to increase, it is crucial to address the challenges associated with the ABC phenomenon and the safety concerns related to administration. This is important for patients who test positive for APAs. While various strategies have been suggested to reduce or lessen the impact of APAs, there is still a lack of strong and universally effective solutions for altogether preventing or eliminating the issue. The following sections outline multiple approaches that mitigate the effects of APAs.

### 5.1. Structural Modifications of PEG and Conjugation Chemistry

#### 5.1.1. Alteration of PEG–Lipid Linkages

**Figure 4:**
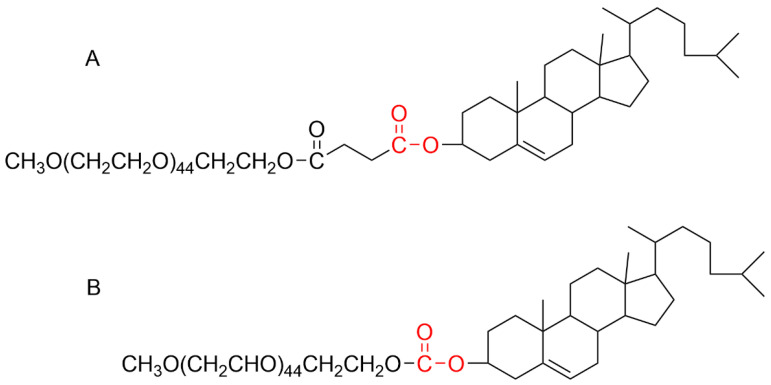
The molecular structure of mPEG-CHEMS (**A**) and mPEG-CHMC (**B**).

Xu et al. [57] synthesized two PEGylated lipid derivatives (mPEG-CHEMS and mPEG-CHMC) to reduce the immunogenicity of PEG groups during multiple dosing (Figure 4). The PEG moiety in these derivatives is linked to a single ester bond, which permits enzymatic cleavage by esterases in the bloodstream, thereby facilitating effective removal of PEG without compromising the integrity of the liposomal membrane. Administration of mPEG-CHMC liposomes in rats led to a mild ABC effect and increased hepatic uptake. Conversely, repeated injections of mPEG-CHEMS liposomes did not trigger the ABC phenomenon or increase hepatic accumulation.

#### 5.1.2. Shedding of PEG Chains

Antibody responses to PEGylated drugs are primarily directed against the PEG moiety. Judge et al. [58] reported that using rapidly dissociable PEG–lipids from the liposome membrane could mitigate APA reactions and prevent APA interactions with PEGylated liposomes in a murine model. They modified PEGylated liposomes encapsulating plasmid DNA (pDNA) with short-acyl-chain PEG–lipids that easily detached from the membrane. Repeated administration in mice did not reduce the pDNA–liposome gene expression.

Suzuki et al. [50] investigated how the PEG shedding rate impacts APA production and ABC phenomenon incidence following repeated administration of PEGylated LNPs. They found that LNPs modified with short-acyl-chain (fast-shedding) PEG–lipids induced significantly fewer APAs than those with long-acyl-chain (slow-shedding) PEG–lipids. In mice, the administration of siRNA-loaded LNPs containing slow-shedding PEG initiated strong APA production and significant ABC effects, resulting in a total loss of activity with subsequent doses. On the other hand, siRNA LNPs featuring fast-shedding PEG maintained the efficacy of later doses. While these LNPs did accumulate more in the spleen, the remaining PEG levels were inadequate to stimulate marginal zone B cells (MZ-B cells). Thus, rapid detachment of PEG from the nanoparticle surface diminishes the immune response against PEGylated liposomes and LNPs.

#### 5.1.3. Modification of PEG Terminal Groups

**Figure 5:**
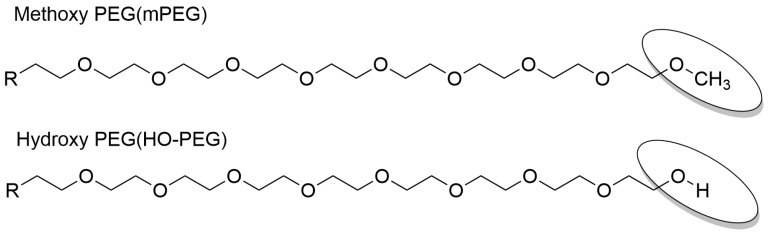
The molecular partial structures of mPEG and HO-PEG.

Sherman et al. [29] investigated the advantages of using hydroxy-PEG (HO-PEG) instead of methoxy-PEG (mPEG) in PEG conjugates synthesis for proteins and other therapeutics (Figure 5). In an ELISA using mPEG-conjugated superoxide dismutase (SOD) as the antigen, they compared the affinity of rabbit serum antibodies against multiple PEGylated conjugates, based on porcine uricase, human serum albumin, or human interferon-α for mPEG and HO-PEG. The results demonstrated that antibodies induced by HO-PEG conjugates had a geometric mean APA titer ratio of 1.1 (range: 0.9–1.5) when tested against mPEG-SOD. In contrast, the antibodies produced by mPEG conjugates demonstrated a notably higher relative titer, with a geometric mean of 3.0 (range: 1.1–20), suggesting enhanced immunoreactivity toward mPEG.

Analysis of 2074 blood samples from Shanghai [59] found a significant difference in seropositivity rates. IgM antibodies against mPEG were detected in 17.98% of samples, compared to only 1.11% for HO-PEG, proposing that HO-PEG modified drugs have lower immunoreactivity when APAs pre-exist. Zhan Changyou’s team at Fudan University further showed that HO-PEG modified with various hydrophobic groups or different PEG chain lengths could avoid detection by pre-existing APAs in clinical environments. HO-PEG can escape APA-mediated capture, reducing macrophage phagocytosis and enhancing the stability of nucleic acid therapeutics [60].

### 5.2. Combination with Immunomodulators

Studies have shown that co-administration of immunomodulatory agents, such as methotrexate, mycophenolate mofetil, azathioprine, and leflunomide, significantly enhances the therapeutic response to PEGylated uricase (Pegloticase) [61]. Pegloticase is used to lower serum uric acid levels, while the development of APAs will limit its efficacy in many patients. An article reviewed the overall response rate of Pegloticase co-administration with immunomodulators in 82 cases across 10 studies. The overall response rate for Pegloticase combined with immunomodulators and alone was 82.9% and 42%, respectively.

The response rates by individual immunomodulators for combination therapy were 87.5% (35 of 40 patients) for Methotrexate, 86.4% (19 of 22) for Mycophenolate mofetil, 63.6% (7 of 11) for Azathioprine, and 66.7% (4 of 6) for Leflunomide. These findings suggest that combining Pegloticase with immunosuppressive agents can significantly improve treatment outcomes in patients with uncontrolled gout. Furthermore, using immunosuppressants alongside Pegloticase may lower the risk of infusion-related reactions during treatment by enhancing its tolerability and safety profile.

### 5.3. Adjustment of Dosing Regimen

One simple clinical strategy to reduce the effect of APAs is to extend the interval between doses because of the time-dependent nature of responses. However, maintaining effective plasma drug concentrations also depends on the timing between doses. Therefore, the design of appropriate dosing intervals in clinical practice should carefully balance clinical efficacy and immunological risk. Numerous studies indicate that administering large doses of PEGylated nanocarriers and extending the time between injections can significantly lower the occurrence of the ABC phenomenon [62,63]. However, higher doses may also pose risks of severe side effects or affect the therapeutic efficacy of the encapsulated drug.

### 5.4. Development of Alternative Polymers

**Figure 6:**
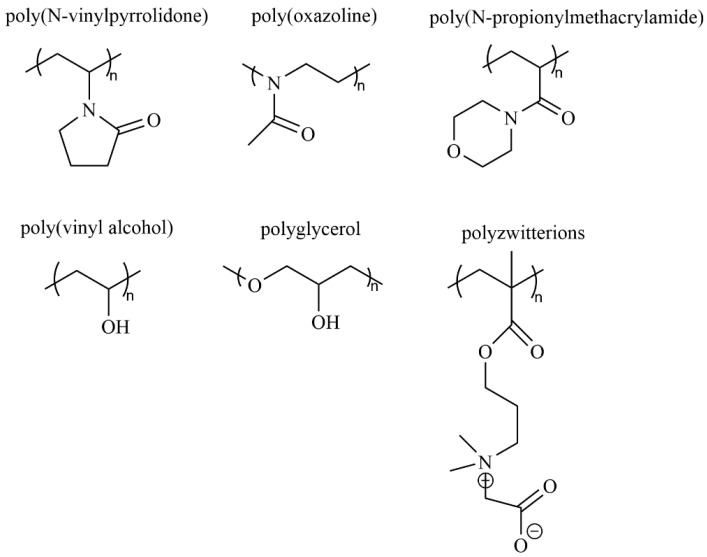
Chemical structures of various synthetic polymers for protein-polymer conjugation (redrawn and integrated from Refs [64,65,66,67,68]).

One effective strategy to mitigate the impact of APAs is to identify a compatible polymer with physicochemical properties and pharmacokinetic advantages similar to PEG, but with reduced immunogenicity [19]. Researchers are highly interested in exploring a variety of natural and synthetic hydrophilic and zwitterionic polymers [64], including poly(N-vinylpyrrolidone) [65], poly(oxazoline) [66], poly(N-propionylmethacrylamide) [67], poly(vinyl alcohol) [68], polyglycerol [55], and polyzwitterions [64]. These candidates are evaluated for their potential to conjugate with proteins and function similarly to PEG (Figure 6). Some candidates have demonstrated promising abilities to reduce immunogenicity and circumvent the ABC phenomenon.

Shin K and colleagues [69] found that liposomes modified with polyglycerol (PG) did not induce anti-polymer immune responses or the ABC phenomenon upon repeated administration. In tumor mouse models, these PG-based liposomes also improved the therapeutic effectiveness of encapsulated doxorubicin. The researchers suggested that the hydroxymethyl side chains in PG repeating units -(O-CH_2_-CH(CH_2_OH))_n_- create steric hindrance, which obstructs effective binding to surface immunoglobulins on reactive splenic B cells and hampers specific B cell activation. This could potentially decrease the production of anti-PG IgM, a crucial initiator of the ABC phenomenon.

Romberg et al. [70] reported that liposomes coated with poly(hydroxyethyl-L-glutamine) or poly(hydroxyethyl-L-asparagine) exhibited stealth-like properties comparable to PEGylated liposomes and were less prone to inducing the ABC effect. They noted that the ABC phenomenon still occurred when these liposomes were administered at low doses. Ishihara et al. [71] demonstrated that nanoparticles coated with poly(N-vinyl-2-pyrrolidone) (PVP) showed prolonged circulation in rats after the initial dose, although the half-life was shorter than that of PEGylated nanoparticles. Importantly, due to reduced anti-PVP IgM antibodies, repeated injections of PVP-coated nanoparticles at various intervals did not trigger the ABC effect. Despite these advancements in identifying PEG alternatives, challenges persist. These potential polymers still encounter limitations regarding biocompatibility, drug delivery performance, stability, production cost, and broad applicability. Further research is required to identify a universally accepted substitute for PEG.

### 5.5. Mild Grafting Reactions for PEG and Alternative Polymers

In addition to the structural properties of polymers themselves, the chemical methods used to graft PEG and alternative polymers onto therapeutic agents play a crucial role in determining conjugate performance. A variety of mild and biocompatible conjugation strategies have been developed to enable site-selective and stable linkage formation under physiological or near-neutral conditions. Among the most commonly used reactions are NHS ester–amine coupling, which allows for efficient conjugation to lysine residues; maleimide–thiol addition, which enables selective modification of cysteine-containing biomolecules; and azide–alkyne cycloaddition (“click chemistry”), which provides high-yield, orthogonal conjugation without interfering with native biological functionalities. Other approaches, such as oxime or hydrazone ligation, allow for pH-responsive or reversible conjugates, while enzyme-mediated ligation systems (e.g., sortase or transglutaminase-based) offer precise control over attachment sites. These chemistries have not only been applied in conventional PEGylation but are also increasingly adopted in the design of next-generation stealth carriers using polyoxazolines, polyglycerols, and zwitterionic polymers. A recent study by Wang et al. highlights the use of well-defined grafting strategies to construct degradable, functional polymer–drug conjugates with improved therapeutic profiles and reduced immunogenicity [72].

### 5.6. Antibody Neutralization Strategies

Antibody neutralization strategies provide direct intervention against anti-PEG antibodies. Pre-administration of empty PEGylated liposomes or high-molecular-weight free PEG can saturate existing APAs. McSweeney et al. [73] demonstrated that pre-infusion of 40 kDa free PEG effectively extended the circulation time of PEGylated liposomal doxorubicin (PLD) in mice to 48 h. In contrast, free PEG with a molecular weight of 10 kDa or less showed minimal effect. Importantly, administering free PEG to mice that PEGylated liposomes had previously sensitized did not trigger any hypersensitivity reactions. Additionally, even in mice with pre-existing APAs, the treatment with free PEG did not result in an exaggerated antibody response.

A comparative summary of major PEG modification strategies and their reported effects on immunogenicity is provided in Table 2, highlighting how different approaches impact anti-PEG antibody responses through structural or pharmacological means.

## 6. Conclusions and Perspectives

Current research shows that the generation of APAs is closely linked to an individual’s genetic background, immune status, and prior exposure history. The mechanisms involved include interactions between marginal zone B cells, follicular B cells, and other immune cell populations. Moreover, because PEG is widely used in various personal care products and other sources, many individuals may already have pre-existing APAs in their bodies. However, due to the lack of standardized and validated detection methods, the exact prevalence and concentration of anti-PEG IgM and IgG in healthy individuals need further investigation.

The presence and levels of APAs, whether caused by treatment or existing beforehand, are closely related to the pharmacokinetic characteristics of PEGylated therapies, such as biodistribution, clearance, and half-life. Anti-PEG antibodies can significantly reduce therapeutic effectiveness by hastening drug elimination. Furthermore, the presence of these antibodies may heighten the likelihood of serious adverse reactions, particularly in extended treatment courses, where infusion-related incidents or other immune-mediated effects may arise. Therefore, sensitive detection of APAs before, during, and after therapy may be critical for ensuring the safety and efficacy of PEGylated treatments. In preclinical studies, animals should be screened for pre-existing APAs before the study begins. For animals that test positive for APAs, integrating area under the curve (AUC), toxicokinetics (TK)/pharmacokinetics (PK) data, anti-drug antibody (ADA) responses, and neutralizing antibody assays is essential for assessing the impacts on drug exposure and toxicity. Likewise, clinical trial designs should incorporate baseline screening for APAs and carefully document patients’ prior treatment histories, as previous exposure to PEGylated agents may affect responses to subsequent therapies.

As a result, devising strategies to lessen APA production and diminish their biological impacts has emerged as a primary concern. While recent studies have notably enhanced our comprehension of APA-related immune reactions, additional research is essential to clarify the exact mechanisms of APA generation, to create more sensitive and specific detection methods, and to formulate effective strategies for suppressing or circumventing APA responses. Ultimately, with continued progress in this field, we can expect to better understand and manage the clinical impact of anti-PEG antibodies, thereby optimizing the therapeutic applications of PEGylated drugs.

Future research should focus on several key areas. First, there is a pressing need to elucidate the precise immunological mechanisms behind APA generation, including genetic and environmental factors that predispose individuals to antibody formation. Second, the development of standardized, sensitive, and specific detection methods for APA—particularly those that distinguish between IgG, IgM, and IgE, which is essential for both preclinical and clinical monitoring. Third, establishing screening guidelines and decision-making frameworks for APA-positive patients before PEGylated therapy will be critical for personalized treatment. Finally, further exploration and validation of alternative stealth polymers with low immunogenicity, such as polyglycerols or poly(oxazolines), are necessary to replace PEG in next-generation drug delivery systems. Addressing these gaps will be vital for overcoming current limitations and enhancing the long-term safety and efficacy of PEGylated therapeutics.

## Figures and Tables

**Table 2:** Summary of PEG modification strategies and their effects on immunogenicity.

Modification Strategy	Mechanism and Example	Effect on Immunogenicity	Ref.
Cleavable PEG–lipid linkages	mPEG-CHEMS, cleaved by esterases	Reduced ABC effect; increased liver uptake	[57]
Fast-shedding PEG–lipids	Short-acyl chain PEG-lipids	Significantly reduces APA induction and ABC phenomenon	[50,58]
Terminal group substitution	HO-PEG vs. mPEG	HO-PEG shows lower APA recognition; reduced immunogenicity	[29]
Combination with Immunomodulators	PEGylated uricase + Methotrexate	Inhibits APA formation; improves therapeutic response	[61]
Use of alternative polymers	Polyglycerol, polyoxazoline, PVP, etc.	Avoids APA entirely; shows comparable stealth properties	[65,66,67,68,69,70,71]
Antibody neutralization with free PEG	Pre-infusion of 40 kDa free PEG	Temporarily blocks APAs; restores circulation time	[73]

## Abbreviations

The following abbreviations are used in this manuscript:

PEG	Polyethylene glycol
APAs	Anti-PEG antibodies
MPS	Mononuclear phagocyte system
RES	Reticuloendothelial system
ELISA	Enzyme-linked immunosorbent assay
BCRs	B cell receptors
TFH	Follicular helper T
MHC	Major histocompatibility complex
TI-2	Thymus-independent type 2
TNF	Tumor necrosis factor
ABC	Accelerated blood clearance
HSRs	Hypersensitivity reactions
CARPA	Complement activation-related pseudoallergy
PHA	Passive hemagglutination assay
WB	Western blot
AMMP	Acoustic membrane microparticle technology
SPR	Surface plasmon resonance
MSD	Meso scale discovery
DCBA	Dual cytometric bead assay
PG	Polyglycerol
PVP	Poly(N-vinyl-2-pyrrolidone)
